# Genetic Therapies for Retinitis Pigmentosa: Current Breakthroughs and Future Directions

**DOI:** 10.3390/jcm14165661

**Published:** 2025-08-11

**Authors:** Zofia Pniakowska, Natasza Dzieża, Natalia Kustosik, Aleksandra Przybylak, Piotr Jurowski

**Affiliations:** 1 Department of Ophthalmology and Vision Rehabilitation, Medical University of Lodz, 90-549 Lodz, Poland; natasza.dzieza@stud.umed.lodz.pl (N.D.); natalia.kustosik@stud.umed.lodz.pl (N.K.); aleksandra.przybylak@stud.umed.lodz.pl (A.P.); piotr.jurowski@umed.lodz.pl (P.J.); 2 Optegra Eye Clinic, 90-127 Lodz, Poland

**Keywords:** gene therapy, retinitis pigmentosa, inherited retinal diseases, Luxturna, OCU400, CRISPR, MCO-010, QR-1123, SPVN06, ophthalmology

## Abstract

Retinitis pigmentosa is a group of inherited retinal dystrophies characterized by progressive photoreceptor cell loss leading to irreversible vision loss. Affecting approximately 1 in 4000 individuals worldwide, retinitis pigmentosa exhibits significant genetic heterogeneity, with mutations in genes such as *RHO*, *PRPF31*, *RPE65*, *USH2A*, and *NR2E3*, which contribute to its diverse clinical presentation. This review outlines the genetic basis of retinitis pigmentosa and explores cutting-edge gene-based therapeutic strategies. Luxturna (voretigene neparvovec-rzyl), the first FDA-approved gene therapy targeting *RPE65* mutations, represents a milestone in precision ophthalmology, while OCU400 is a gene-independent therapy that uses a modified *NR2E3* construct to modulate retinal homeostasis across different RP genotypes. Additionally, CRISPR–Cas genome-editing technologies offer future potential for the personalized correction of specific mutations, though concerns about off-target effects and delivery challenges remain. The article also highlights MCO-010, a novel optogenetic therapy that bypasses defective phototransduction pathways, showing promise for patients regardless of their genetic profile. Moreover, QR-1123, a mutation-specific antisense oligonucleotide targeting the P23H variant in the *RHO* gene, is under clinical investigation for autosomal dominant RP and has shown encouraging preclinical results in reducing toxic protein accumulation and preserving photoreceptors. SPVN06, another promising candidate, is a mutation-agnostic gene therapy delivering RdCVF and RdCVFL via AAV to support cone viability and delay degeneration, currently being evaluated in a multicenter Phase I/II trial for patients with various rod–cone dystrophies. Collectively, these advances illustrate the transition from symptom management toward targeted, mutation-specific therapies, marking a major advancement in the treatment of RP and inherited retinal diseases.

## 1. Introduction

Retinitis pigmentosa (RP) is a group of hereditary diseases that significantly affect patients’ quality of life, primarily through progressive vision loss, often leading to visual field constriction and eventual blindness. Affecting over 1.5 million people globally, mainly individuals under the age of 60, RP is one of the most widespread forms of inherited retinal disease (IRD), with an estimated global prevalence of approximately 1 in 4000 [1].

In RP, the earliest changes occur in rod photoreceptors, which gradually degenerate before cone cells are affected. Night blindness often emerges as the first noticeable symptom, eventually giving way to a shrinking visual field. Despite the progressive nature of the condition, central vision—mediated by the macula—tends to remain largely unaffected until the disease reaches more advanced stages. Typical ophthalmoscopic findings include pigment deposits resembling bone spicules, primarily located in the peripheral or mid-peripheral retina, thinning of the retinal blood vessels, and a pale, wax-like appearance of the optic disc [2].

RP can be classified based on its mode of inheritance into autosomal dominant (15–25% of cases), autosomal recessive (the most common form, affecting 50–60%), X-linked (5–15%), and sporadic cases (about 40–50%) [3]. These categories may overlap. The pattern of inheritance is critical to define, as it directly influences the age of symptom onset. The X-linked recessive form has a more severe clinical course, with possible visual impairment emerging around the fourth decade of life, whereas in autosomal dominant RP, symptoms typically appear around the sixth decade [4]. Consequently, identifying the inheritance pattern and evaluating the genetic risk of RP is essential; therefore, genetic testing should be encouraged, and a comprehensive family history should be taken.

Retinitis pigmentosa can also be divided into non-syndromic and syndromic forms. The first one includes cases affecting the retina without involving other systems or organs [1], while the second one includes conditions like Usher syndrome or some forms of Leber congenital amaurosis (LCA).

Three stages of the disease—early, mid, and late—can be distinguished based on the clinical picture. In the early stage, night blindness (nyctalopia) is the leading symptom, along with difficulty adapting to low-light environments. Frequently, nyctalopia does not significantly affect patients’ quality of life, leading to delayed diagnosis until more advanced stages [5].

Nyctalopia that manifests early in the disease typically escalates during the mid-stage, affecting daily functioning. Alongside the appearance of pigment migration from the retinal pigment epithelium (RPE), which forms bone-spicule deposits in the peripheral retinal area and leads to retinal degeneration, patients experience difficulties such as impaired night driving ability or increased accidents due to poor visibility [6]. In the late stage, cone cell damage results in a marked reduction in visual acuity [7], and pigment is deposited throughout the retina, including the macula [8]. As a result, patients experience significant visual field constriction and tunnel vision [1], making independent navigation increasingly difficult [9].

## 2. Methods Section

This review aims to summarize current and especially emerging therapeutic strategies for RP, with a particular emphasis on gene-based and optogenetic approaches. Evidence was synthesized from peer-reviewed literature and data from clinical trials published before June 2025. A comprehensive search was conducted using the PubMed, Scopus, Web of Science, Google Scholar, and ClinicalTrials.gov databases.

Examples of search terms used include the following: “retinitis pigmentosa”, “inherited retinal diseases”, “gene therapy”, “optogenetics”, “Luxturna”, “OCU400”, “CRISPR”, “MCO-010”, “QR-1123”, and “SPVN06”. Boolean operators (AND, OR) were applied to refine the search. Searches were limited to English-language publications.

Additional references were identified through manual screening of bibliographies from key articles and relevant reviews. The inclusion criteria focused on peer-reviewed studies, including original research, clinical trials, systematic reviews, meta-analyses, and authoritative clinical summaries (e.g., GeneReviews, StatPearls). Press releases and regulatory documents were included only when peer-reviewed publications were not available and were clearly indicated in the manuscript. Editorials, opinion pieces, and non-English texts were excluded.

## 3. Brief Characteristics of Selected Genes Causing RP

### 3.1. Rhodopsin Gene (RHO)

Rhodopsin is a protein that constitutes over 90% of the protein content within the rod outer segment (ROS) disc and is essential for low-light vision [10]. It is a light-sensitive molecule composed of 348 amino acids, synthesized from the *RHO* gene, which includes five coding exons. It is purely expressed in rod photoreceptors and is a part of the large family of G-protein-coupled receptors, characterized by seven transmembrane helices. Structurally, rhodopsin may be divided into three domains: the intradiscal region, the transmembrane as a center, and the cytoplasmic portion [11]. Mutations in the gene encoding rhodopsin are responsible for approximately 26.5% of all diagnosed cases of autosomal dominant RP and 10% of all RP [12].

The *RHO* gene contains characteristic mutational hot spots at amino acid positions 135, 190, and 347, where mutations are associated with various forms of RP across the population [13]. There are also known variations within the cytoplasmic domain of the gene; for instance, the *Pro347Leu* mutation in the *RHO* gene is associated with more serious clinical symptoms [14]. Nevertheless, all of these mutations lead to the same outcome: degeneration of rod cells, ultimately resulting in RP.

### 3.2. PRPF31

The *PRPF31* gene is the most frequently implicated splicing factor in autosomal dominant retinitis pigmentosa [15]. Splicing factors are crucial in retinal development and the maintenance of visual function [16]. Although these factors regulate the splicing process in all cell types, pathogenic mutations in their encoding genes appear to affect only the retina, which—together with high *PRPF31* expression in the retina—reveals the retina’s critical dependence on alternative splicing [15].

The proper function of *PRPF31* is important for maintaining cell survival, mainly due to its role in assembling the U4/U6.U5 tri-small nuclear ribonucleoprotein (tri-snRNP) complex. When *PRPF31* is absent or deleted, the formation of this tri-snRNP complex is disrupted, ultimately stopping the splicing of precursor mRNA (pre-mRNA). *PRPF31*, along with *PRPF6*, simplifies the integration of the U4/U6 di-snRNP with the U5 snRNP during each splicing cycle, enabling the completion of the tri-snRNP necessary for its function [17].

Over 100 different genetic variants have been identified in the *PRPF31* gene [18]. The pathogenic mechanism underlying these mutations is haploinsufficiency, which also causes incomplete penetrance of RP symptoms [19]. Clinical observations indicate that patients with *PRPF31* mutations often become blind by their third decade of life [20].

### 3.3. RPE65

The *RPE65* gene encodes the *RPE65* enzyme, which plays an essential role as an isomerase, mediating the isomerization of all-trans-retinyl ester to 11-cis-retinol. The absence of *RPE65* leads to reduced levels of 11-cis-retinal and the accumulation of retinyl esters in the retinal pigment epithelium [21]. *RPE65* is found in RPE and cone photoreceptors apart from the rods [22]; however, its role in the cones has not been fully identified yet. There is a hypothesis that, in addition to the conventional retinoid cycle utilized by rods, cones may rely on a distinct visual cycle [7], likely dependent on the *RPE65* enzymatic activity. These findings indicate that *RPE65* is critical for the function of both rod and cone photoreceptors.

From a clinical perspective, mutations in the *RPE65* gene result in nyctalopia, poor pupillary light response, nystagmus, reduced central vision acuity, and the oculo-digital sign—for instance, rubbing or pressing the eye to trigger phosphenes and create a perception of light [23].

### 3.4. NR2E3

*NR2E3* is essential for the development of the rod photoreceptors, in which it is expressed [24]. Mutations in this gene lead to disruption of normal retinal architecture, including the absence of rhodopsin expression and an increased number of cone photoreceptors, which are mislocalized within retinal layers typically occupied by rod cells [25].

A mutation in the *NR2E3* gene is one of the causes of autosomal dominant RP and results in a characteristic phenotype, such as degeneration of rod photoreceptors, eventually leading to cone dysfunction. There are some clinical symptoms related to the *NR2E3* mutation, including nyctalopia that occurs in adolescence or early twenties, visual field impairment, which starts around the third decade, and a decline in visual acuity that begins in the fifth decade of life [26].

## 4. Current Methods of Prevention and Treatment

Retinitis pigmentosa is a degenerative retinal disorder for which no definitive cure currently exists. Modern therapeutic approaches are primarily focused on slowing disease progression and improving patients’ quality of life.

### 4.1. Diet (Vitamin A; Omega-3 Fatty Acid; Lutein; Zeaxanthin)

Vitamin A palmitate is one of the most significant antioxidant components used in the treatment of RP. It plays a crucial role in growth, development, immune response, epithelial cell integrity, and proper visual function [27]. In the retina, vitamin A is necessary for the conversion of 11-cis-retinal to all-trans-retinal within the visual cycle, a process that occurs under the influence of light and activates rhodopsin, thereby initiating the phototransduction cascade.

The antioxidant and neurotrophic effects of vitamin A may help preserve photoreceptors. Long-term supplementation of this vitamin at doses of 15,000 IU per day has been shown in former studies to slow the progression of photoreceptor loss in the retina [28]. Currently, however, there is no standardization for vitamin A treatment due to conflicting results in clinical trials [29]. The liver serves as the primary storage site for vitamin A; therefore, when supplementation is considered, regular monitoring of serum retinol levels, triglyceride concentrations, and hepatic enzyme activity (aspartate aminotransferase, alanine aminotransferase, and alkaline phosphatase) is essential.

Other dietary components, such as omega-3 fatty acids, lutein, and zeaxanthin, are also potential elements for maintaining retinal viability and delaying the progression of degenerative processes. Omega-3 fatty acids may enhance retinal protection by reducing inflammation and supporting retinal cell health, while lutein and zeaxanthin further contribute to retinal integrity by minimizing oxidative damage [30].

### 4.2. Pharmacological Approach in Cystoid Macular Edema (CME)

In approximately 38% of RP cases, CME appears as a common manifestation, which can result in reduced visual acuity [31]. It has been proven that the first-line treatment for this complication includes topical and oral carbonic anhydrase inhibitors. Second-line therapies, which have also demonstrated effectiveness, include intravitreal steroid injections, oral corticosteroids, anti-VEGF injections, and topical or locally administered non-steroidal anti-inflammatory drugs [32].

### 4.3. Surgical Treatment—ARGUS II

ARGUS II is a prosthesis that involves the implantation of an epiretinal electrode chip, which induces electrical impulses in the retina. Patients must wear glasses equipped with a video recorder that converts images to electrical impulses, enabling them to see the lines and edges of surrounding objects [33]. Despite satisfactory results in terms of visual function, approximately 30% of patients experienced adverse effects, including conjunctival erosion, conjunctival separation, low intraocular pressure, and ocular infections. Moreover, ARGUS II is used only for patients with very minimal residual light reception and end-stage RP, meaning that individuals with a less advanced form of RP cannot apply this therapeutic method [34].

Unfortunately, ARGUS II is no longer available to new patients due to its discontinuation, although some patients still have the device implanted, and repair or technical assistance is now limited or unavailable.

### 4.4. Hyperbaric Oxygen

The retina is a highly metabolically active tissue with photoreceptors particularly dependent on efficient oxygen delivery to sustain their function [35]. It has been proven that transient hyperoxic conditions—as provided during the hyperbaric oxygen therapy, which exposes patients to barometric pressure above sea level and enhances the oxygen diffusion into tissues—may support the survival of the photoreceptors by helping to meet their metabolic demands. Although hyperbaric oxygen therapy has not yet been sufficiently researched, a few studies have demonstrated that patients undergoing this therapy, applied with a time-varying, predetermined frequency, experienced recovery of the electroretinography (ERG) b-wave amplitude [36]. However, apart from beneficial results, this therapy resulted in increased levels of reactive oxygen metabolites, leading to cone cell death [37], keratoconus, cataracts, and age-related macular degeneration (AMD) [38].

### 4.5. Stem Cells

Stem cells may differentiate into any type of human cell; therefore, this therapy is based on the conversion of these cells into retinal cells and the formation of new synapses [39]. The main sources are bone marrow-derived stem cells, embryonic stem cells, and induced pluripotent stem cells [40]. In 2015, a clinical trial was conducted involving an intravitreal injection; after 3 months of this therapy, the patients’ vision improved. However, this improvement deteriorated over the following 12 months [41]. Despite the promising concept, other studies have shown that stem cell therapy may cause intraocular lens (IOL) displacement, likely due to traction forces near the place of injection and the formation of connective tissue in the vitreous cavity, ultimately leading to retinal detachment [42,43].

## 5. Emerging Genetic Therapies for RP

### 5.1. Gene Therapy and Delivery Methods

Gene therapy is considered one of the most promising approaches for treating RP, aiming to correct or compensate for genetic mutations underlying the disease. Viral vectors, such as adeno-associated virus (AAV), are commonly used for in vivo delivery of genetic material; however, they are limited by their small cargo capacity and do not integrate into the host genome. Lentiviruses, although capable of carrying larger genetic fragments, may randomly integrate into DNA, posing a risk of oncogenesis.

As an alternative to viral methods, non-viral systems—such as lipid-based nanocarriers, polymer nanoparticles, and exosomes—have shown promising results in preclinical studies. Each of these methods has its own advantages and limitations, and the choice depends on the specific application. Ongoing research aimed at improving delivery precision and minimizing side effects offers hope for the development of tissue-specific vectors and effective in vivo gene therapies in the future [44,45].

### 5.2. Luxturna

#### 5.2.1. What Exactly Is Luxturna?

**Figure 1:**
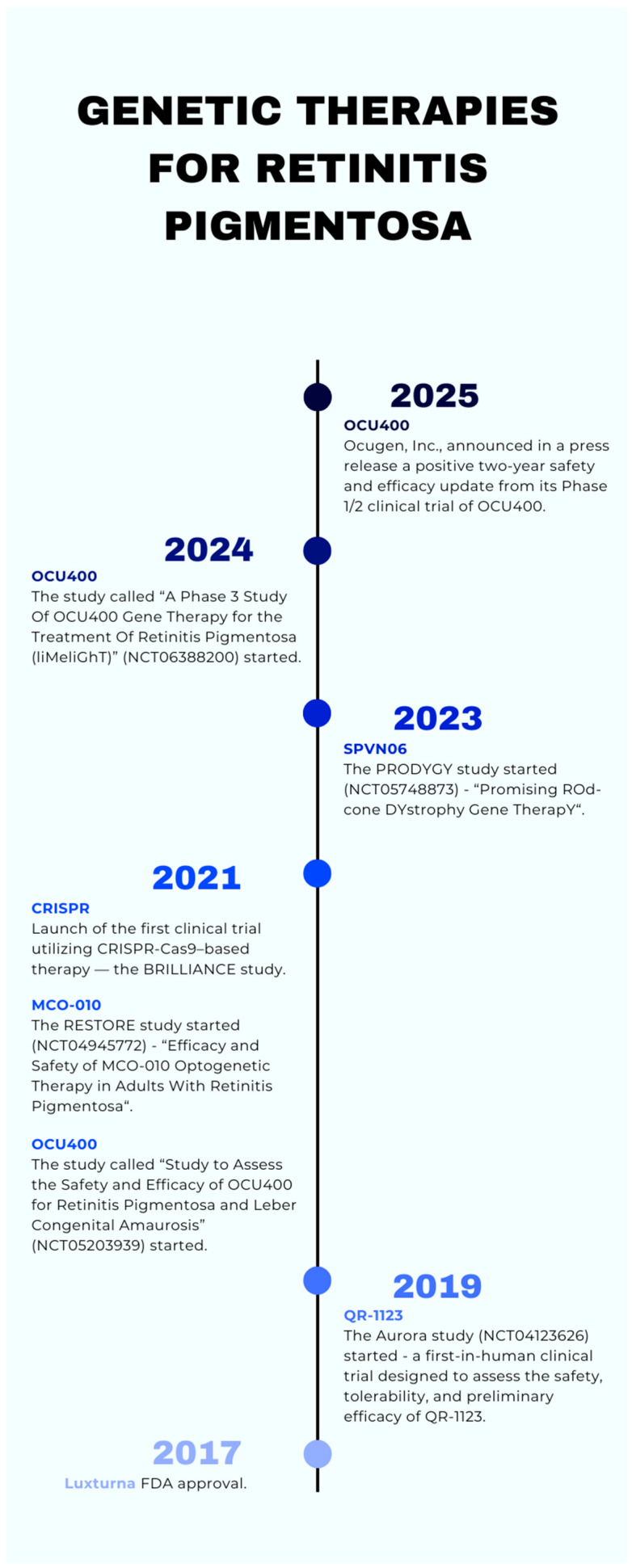
Timeline of genetic therapies for retinitis pigmentosa [46,57,58,59,61,65,68,70].

Luxturna (voretigene neparvovec-rzyl) is the first FDA-approved gene therapy for an inherited retinal disease, representing a significant milestone in the treatment of vision loss. This medicine was approved on 19 December 2017 and is manufactured by Spark Therapeutics, Inc. It is administered as a subretinal injection in the form of a suspension and is specifically indicated for patients with confirmed biallelic *RPE65* mutation-associated retinal dystrophy, including certain forms of RP [46] (Figure 1).

Luxturna works by delivering a correct copy of the *RPE65* gene directly into the retinal cells, using an AAV as a carrier. After Luxturna is injected, the virus incorporates the functional gene into the cells, allowing them to produce the missing *RPE65* enzyme, which is necessary for physiological visual function. The virus is non-pathogenic to humans. To reduce the risk of immune rejection of the treatment, patients receive immunosuppressive therapy three days before the first injection, continue it during treatment of both eyes, and maintain it for 14 days after the second injection. Each eye is treated separately, with at least a six-day interval between procedures [47] (Table 1).

#### 5.2.2. Safety Profile and Adverse Effects of Luxturna

The manufacturer—Spark Therapeutics, Inc.—outlines several important precautions associated with Luxturna’s use. There is a potential risk of infection, such as endophthalmitis, as well as the possibility of permanent vision loss and retinal complications, including macular holes, retinal tears, or detachment—all of which require careful monitoring. Patients are advised to avoid air travel and altitude changes until intraocular air bubbles have resolved, as these may increase the risk of irreversible vision loss. Inflammation, including vitritis, has also been observed; however, it typically resolves with corticosteroid treatment without long-term impact on vision [48] (Table 1). According to the European Medicines Agency, the most frequent side effects of Luxturna (occurring in more than 1 in 20 patients) include conjunctival hyperemia, cataract, and elevated intraocular pressure [47].

In a comprehensive systematic review, researchers searched MEDLINE, Embase, the Cochrane Central Register of Controlled Trials, and ClinicalTrials.gov. The initial search was completed on 8 May 2020 and subsequently updated on 24 May 2022, incorporating references from relevant trials and reviews. Out of 3548 identified records, 80 publications met the eligibility criteria, covering 28 registered clinical trials and 5 post-market surveillance studies investigating AAV-mediated gene therapy for different retinal diseases, including RP. The analyzed studies included controlled or single-arm interventional trials. The primary focus was on safety, assessing ocular adverse events such as intraocular inflammation, cataract formation, retinal detachment, and elevated intraocular pressure, alongside secondary effects such as eye pain, changes in vision, and pupillary responses. The secondary outcomes assessed the efficacy of gene therapy—improvement in best-corrected visual acuity (BCVA) and visual sensitivity—as well as systemic effects, including viral dissemination and immune activation. The findings showed a cumulative incidence of serious adverse events (SAEs) at 8%, predominantly linked to the surgical procedure rather than the gene therapy vector itself. Systemic immune responses were observed in 31% of patients, and improvements in BCVA and visual sensitivity were reported in 41% and 51% of patients, respectively. However, heterogeneous adverse event reporting limited the robustness of the conclusions in this comprehensive systematic review [49] (Table 1).

The PERCEIVE study is an ongoing, multicenter, observational study and currently the largest trial assessing the real-world safety and effectiveness of voretigene neparvovec-rzyl in patients with biallelic *RPE65*-associated IRDs. Among 103 enrolled patients (mean age: 19.5 years; follow-up periods of up to 2.3 years), 34% experienced ocular treatment-emergent adverse events (TEAEs), and the most frequent was mild chorioretinal atrophy (12.6%). Ocular TEAEs of special interest were observed in 17.5% of patients, including intraocular inflammation (6.8%), elevated intraocular pressure (4.9%), and foveal thinning (3.9%). Non-ocular TEAEs were less frequent; the most common one was headaches. Two serious ocular TEAEs and one steroid-related psychiatric event were reported. Luxturna treatment led to durable improvements in full-field light sensitivity, especially in patients under 18 years. Visual acuity remained largely unchanged. Overall, the findings of the PERCEIVE study are consistent with previous clinical trials, while highlighting chorioretinal atrophy as a newly identified adverse event, which underscores the need for continued long-term monitoring [49] (Table 1).

#### 5.2.3. Key Clinical Trials: Phase 3 Data

The Phase 3 trial “A Phase III Trial of Gene Therapy for LCA Due to *RPE65* Mutations” (ClinicalTrials.gov ID: NCT00999609), sponsored by Spark Therapeutics, Inc., is a multicenter, open-label, randomized controlled trial assessing the efficacy and safety of voretigene neparvovec-rzyl in patients with confirmed biallelic *RPE65* mutation-associated IRD. The inclusion criteria included individuals ≥3 years old, diagnosed with LCA or RP caused by *RPE65* mutations, who had sufficient viable retinal cells and had measurable visual function. The main exclusion criteria included ocular conditions that could bias the interpretation of results, such as previous intraocular surgery within six months, prior exposure to gene therapy, or active ocular infection/inflammation. The trial enrolled 31 participants, randomized at 2:1 to receive subretinal injection of voretigene neparvovec-rzyl or remain in the control group (untreated). The results demonstrated notable improvements in functional vision—measured by the multi-luminance mobility test (MLMT). The treated group showed a mean improvement of 1.8 light levels at one year, whereas the control group exhibited no statistically significant change from baseline. The secondary outcomes included full-field light sensitivity threshold testing (FST), which also improved considerably in the treated participants, indicating enhanced retinal sensitivity to light. The study demonstrated that the therapy can improve functional vision and has a manageable safety profile in eligible patients [50] (Table 1).

Another trial, “Post-Approval Safety Study of Voretigene Neparvovec-rzyl (Luxturna)” (ClinicalTrials.gov ID: NCT04516369), sponsored by Novartis Pharmaceuticals, is an ongoing, multicenter, observational Phase 3 trial designed to monitor the long-term safety and effectiveness of voretigene neparvovec-rzyl in real-world clinical practice. It includes patients with confirmed biallelic *RPE65* mutation-associated inherited retinal dystrophy who meet the approved indications for treatment. The exclusion criteria involve any disqualifying conditions for Luxturna, such as active ocular infections or inflammation, and any conditions that could affect the accuracy of the study evaluations. The interim results show that the safety profile remains consistent with earlier trials, and no new adverse events have been reported. The reported complications (e.g., inflammation, increased IOP) are consistent with known risks, and continued improvements in full-field light sensitivity support sustained therapeutic effect [51] (Table 1).

#### 5.2.4. Vision Testing and Functional Outcomes

Voretigene neparvovec-rzyl has been shown to improve patients’ performance in mobility tests by enhancing their ability to navigate obstacle courses under varying lighting conditions, simulating real-life visual challenges. These improvements occurred even when standard visual tests did not show marked changes, suggesting benefits in functional vision, such as dark adaptation and retinal sensitivity. Younger patients typically showed greater improvements. However, mobility test outcomes can be influenced by learning effects, patient motivation, or caution and, thus, should be interpreted alongside objective measures [52].

Voretigene neparvovec-rzyl aims to improve subjective vision, while classical psychophysical tests like visual acuity or mobility tests depend heavily on patient cooperation and brain interpretation, which can introduce variability. In contrast, objective measures such as ERG, chromatic pupillometry (CPC), and transcorneal electrostimulation provide direct retinal responses without brain processing, offering potentially clearer insights. However, standard full-field ERG is not sensitive enough to detect changes after treatment, since the therapy targets only the central 20° of the retina and the resulting signal would represent just 1–2% of the full-field amplitude—too small for conventional detection. CPC tests showed that treated areas achieved up to 20% of normal pupil constriction, indicating partial retinal function restoration. Despite the limited number of photoreceptors preserved, long-term evidence shows that this partial recovery can result in sustained therapeutic benefits, emphasizing that even a small pool of functional cells may enable meaningful clinical progress [52].

Fundus autofluorescence (FAF) is a valuable imaging tool for IRD, though *RPE65*-related IRDs typically show an extremely weak FAF signal due to low lipofuscin levels in the RPE. Interestingly, after the voretigene neparvovec-rzyl treatment, some patients demonstrated improved FAF imaging, potentially indicating metabolic restoration in rod photoreceptors. Blue FAF imaging, particularly at 30° or 55°, proved more effective than green FAF in detecting early changes and monitoring the progression of chorioretinal atrophy. Although underutilized in pre-approval trials, FAF may be a valuable component of post-treatment monitoring [52].

#### 5.2.5. Impact and Future Directions of Luxturna

The approval of Luxturna was a significant step in the management of IRDs, establishing gene replacement therapy as a viable approach to stop disease progression and preserve visual function. By successfully replacing the defective *RPE65* gene with a functional copy, Luxturna not only provided a groundbreaking treatment for patients with *RPE65*-associated retinal dystrophy but also initiated substantial investment and research into gene therapies for a broader spectrum of IRDs. Numerous trials are now underway or completed for conditions including LCA, RP, choroideremia, achromatopsia, Stargardt disease, Leber hereditary optic neuropathy, and X-linked retinoschisis, broadening future treatment possibilities for these severe disorders [53,54] (Table 1).

#### 5.2.6. Limitations of Luxturna

Luxturna represents a major advancement in gene therapy for inherited retinal diseases, but it comes with several limitations. Its use is only applicable to patients with confirmed biallelic mutations in the *RPE65* gene, restricting its utility to a narrow group of IRD patients. Delivery requires subretinal injection, an invasive surgical procedure with inherent risks such as inflammation or retinal trauma. Furthermore, the treatment cost (approx. USD 850,000 for both eyes) presents a major barrier to access, though insurance coverage may alleviate some of the financial burden in the United States. Importantly, Luxturna does not fully restore normal vision, and some patients may still experience permanent vision loss or central visual acuity reduction after treatment [48].

### 5.3. OCU400

#### 5.3.1. What Exactly Is OCU400?

OCU400 is a gene-agnostic therapy under development for the treatment of RP [55]. Gene-agnostic therapies are designed to act independently of the specific genetic mutation causing IRDs. Instead of targeting a single gene, they focus on common disease mechanisms, offering the potential to treat a wide range of IRD patients. These approaches include retinal cell reprogramming, neuroprotection, immunomodulation, and optogenetics. By modulating common pathways involved in photoreceptor degeneration, such therapies aim to preserve vision regardless of the underlying mutation [56].

In individuals with RP, dysfunction in the *NR2E3* gene network contributes to retinal degeneration. OCU400 targets the *NR2E3* gene, which regulates various physiological processes, such as photoreceptor development and maintenance, metabolism, phototransduction, inflammation, and cell survival. It is designed to restore retinal homeostasis by introducing a functional copy of *NR2E3* using an AAV vector delivered via subretinal injection [55] (Table 1).

#### 5.3.2. Safety Profile, Adverse Effects, and Visual Function Improvement

The Phase 1/2 open-label, multicenter clinical trial of OCU400 reported favorable preliminary safety outcomes. Eighteen participants with mutations in either the *NR2E3* or *RHO* genes received a unilateral subretinal injection of OCU400 with either of three escalating doses. After 12 months of follow-ups, no SAEs related to the gene therapy were observed in the low- and medium-dose groups. In the high-dose and open-enrollment cohorts, two participants experienced SAEs, both attributed to the surgical procedure itself rather than the investigational product. These events resolved within days to weeks without lasting complications. Additional safety monitoring included ophthalmologic examinations and assessments of immune and biochemical markers, none of which revealed significant safety concerns. Overall, these findings suggest that OCU400 was generally well tolerated across different dosing levels, supporting further investigation in larger studies [57] (Figure 1).

In January 2025, the developer, Ocugen, Inc. (Malvern, PA, USA), a biotechnology company specializing in the development of innovative gene and cell therapies, biologics, and vaccines, announced in a press release a positive two-year safety and efficacy update from its Phase 1/2 clinical trial of OCU400. According to the company’s Chief Medical Officer, the two-year data suggest continued safety and potential therapeutic benefit. Improvements in low-light visual acuity—regarded as a sensitive marker of functional vision—were consistent with earlier findings. All evaluable participants (nine out of nine) demonstrated either improvement or preservation of visual function in the treated eye compared to the untreated eye at both the one- and two-year marks. Additionally, 100% of the treated subjects demonstrated improvement or stabilization in mobility performance, as assessed by multi-luminance mobility testing. Importantly, the improvement in visual function was statistically significant (*p* = 0.01), and this effect was observed across patients regardless of their underlying genetic mutation [58] (Figure 1) (Table 1).

#### 5.3.3. Future Development and Direction of OCU400

The FDA has approved an Expanded Access Program (EAP) for Ocugen’s OCU400, enabling treatment access for eligible patients with RP outside of clinical trials. According to company statements, this program is intended for individuals with early-to-advanced stages of RP who retain some functional retinal tissue, including those previously ineligible for clinical trials and participants from the Phase 1/2 trial who may now be eligible for treatment in the second eye. The EAP aims to increase availability ahead of potential regulatory approval. RP is estimated to affect approximately 300,000 individuals in the U.S. and Europe and 1.6 million worldwide [59] (Figure 1) (Table 1).

OCU400 has also received an important regulatory milestone in Europe, as the European Medicines Agency’s Committee (EMA) for Advanced Therapies issued a positive opinion granting the therapy Advanced Therapy Medicinal Product (ATMP) classification. This classification, intended for therapies that offer novel approaches for serious diseases, may help expedite a regulatory review. OCU400 is the first investigational gene therapy from Ocugen to enter Phase 3 development with a broad indication for RP. According to the developer, this regulatory milestone is expected to support further clinical advancement. Subject to ongoing development and regulatory review, the company projects availability in the U.S. and Europe by 2027 [60] (Table 1).

### 5.4. CRISPR

#### 5.4.1. Modern Classification of CRISPR–Cas Systems

CRISPR–Cas systems are divided into 2 classes, comprising a total of 6 types and 33 subtypes. This classification is based on the structure of effector complexes, proteins responsible for cleaving the target genetic material. In gene therapy, Class 2 systems are used, which rely on a single, multidomain effector protein that binds CRISPR RNA (crRNA). This class is further divided into several types, the most important of which are the following: Type II (Cas9), a widely used CRISPR system, in which Cas9 recognizes and precisely cuts target DNA with the help of a short RNA guide (gRNA). Cas9 is widely used in gene therapy, diagnostics, and scientific research. Type V (Cas12) is similar to Cas9 but with additional features, such as the ability to cut DNA while creating single-stranded “sticky ends”. This property enhances editing precision, allowing for more accurate deletions, insertions, and targeted modifications. Type VI (Cas13) is the newest and best-characterized single-effector CRISPR system, which targets single-stranded RNA. Cas13 is an RNA-specific nuclease guided by an RNA molecule. Particularly promising are Cas13d systems, which allow for precise silencing of specific RNA sequences without permanently modifying the genome, which may be beneficial in situations where DNA editing is undesirable [44] (Table 1).

#### 5.4.2. Clinical Trials: Phases 1, 2, and 3

The clinical trial EDIT-101 is an open-label, interventional, multicenter Phase I/II study conducted using a single ascending dose design, aimed at assessing the safety, tolerability, and preliminary efficacy of the therapy in adults and children with LCA10. This disease is associated with the c.2991 + 1655A > G mutation in intron 26 of the *CEP290* gene (known as the IVS26 mutation), which leads to retinal degeneration and progressive vision loss. EDIT-101 utilizes CRISPR–Cas9 technology to remove the defective sequence in the *CEP290* gene and halt retinal degeneration. The treatment involves a single administration of the drug directly under the retina of the affected eye via subretinal injection [61].

The study includes both adults and children, aged 3 to 17 years, with confirmed homozygous or compound heterozygous forms of this mutation. The trial is open-labeled and non-randomized, and the participants are sequentially assigned to five study cohorts, in which three dose levels of EDIT-101 are administered. Initially, adults receive single doses in an ascending scheme: starting with a low dose, followed by a medium dose, and finally a high dose. After a positive safety assessment in adults, treatment can begin in children, who receive either the medium or high dose. Each patient receives only one dose of EDIT-101 through a surgical procedure involving subretinal injection of the drug in the study eye. Participants are then monitored for 12 months to assess the safety of the therapy (including the risk of dose-limiting toxicity) and any changes in their visual function. The efficacy parameters evaluated include changes in BCVA, light sensitivity, pupil response, retinal function, and patients’ quality of life [61].

The BRILLIANCE study, the Phase I/II trial of EDIT-101, included 14 participants—12 adults and 2 children—with the *IVS26* mutation. During the study, a single dose of EDIT-101 was administered subretinally, and the safety and efficacy were assessed over 12 months. No dose-limiting toxicity was observed, and 79% of patients experienced improvement in at least one measured parameter, such as visual acuity, light sensitivity, visual navigation, or quality of life. Clinically meaningful BCVA was observed in approximately 29% of participants, and improvement in light sensitivity was confirmed in 43%. Additionally, several patients reported significant improvements in quality of life, enabling better performance in everyday tasks such as reading the light on a coffee machine or finding their phone. The safety profile was favorable, with most adverse events being mild or moderate and resolving without serious consequences [61] (Figure 1).

In November 2022, the sponsoring company, Editas, paused further recruitment due to limited efficacy observed in patients with the heterozygous mutation. The most pronounced therapeutic effects were seen in patients with the homozygous mutation, which represents a small patient population estimated at about 300 individuals in the U.S. The company is seeking partnerships to continue the program and to redesign future studies to better tailor dosing and patient groups [62].

Although Phase III has not yet formally begun, it is planned to proceed if a supporting partner is secured for a larger, more focused study. Phase III plans include focusing on patients with the homozygous *IVS26* mutation, further evaluating safety and efficacy in a larger population, using refined endpoints such as functional vision improvement and quality of life, and pursuing regulatory approval (FDA, EMA) following positive results. To summarize, early-phase trials, such as EDIT-101 for *CEP290* mutations in Leber congenital amaurosis, illustrate CRISPR–Cas9’s translational potential, although off-target effects and safe in vivo delivery remain significant hurdles [61].

#### 5.4.3. Future Development and Direction of CRISPR

Currently, gene therapies based on CRISPR technology have not yet been approved for routine use in ophthalmology. However, intensive clinical and preclinical studies are underway, showing promising results in retinitis pigmentosa. CRISPR therapy is a modern gene therapy method based on precise genome editing that allows for the modification, deletion, or insertion of DNA fragments at specific locations within a cell’s genome. This technology relies on the use of a guide RNA, a specially designed RNA sequence that directs an enzyme (most commonly Cas9) to the chosen DNA fragment. The Cas9 enzyme, acting as “molecular scissors”, precisely cuts the DNA at the targeted site. After the DNA is cut, the cell activates its natural repair mechanisms, which can lead to various therapeutic outcomes. If the DNA repair is imprecise, it can result in the gene being disabled (knock-out). Alternatively, if appropriate genetic material is provided, a new gene can be inserted (knock-in). CRISPR therapy has great potential in treating many genetic diseases, including retinitis pigmentosa—an inherited retinal disease that leads to gradual vision loss. In the case of RP, CRISPR therapy can be used to repair damaged genes such as *RHO* or *CEP290*, which are among the most common causes of the disease. Preclinical and clinical studies on the application of CRISPR in RP have shown promising results, including improved vision function in patients with genetic mutations. This makes CRISPR one of the most promising therapeutic tools in ophthalmology, offering hope for treating diseases that were previously considered incurable [62,63] (Table 1).

#### 5.4.4. Off-Side Effects and Challenges of CRISPR

One of the greatest challenges to the widespread clinical application of CRISPR technology is minimizing the risk of unintended genetic changes (off-target effects) outside of the target site. Although more precise Cas9 variants have been developed and guide RNA design has been optimized, the need to introduce double-strand breaks (DSBs) in DNA still carries the risk of cell death, large deletions, or chromosomal rearrangements, which may lead to cancer. For this reason, techniques that do not require DSBs are being intensively developed, such as base editing, prime editing, and their derivatives TWIN-PE and PASTE. These methods enable precise modifications—from single nucleotide changes to larger insertions—to be investigated to reduce reliance on gene therapy. Efficient and safe delivery of the CRISPR system to target cells and tissues remains one of the key challenges in gene therapy. It requires the precise introduction of components such as Cas9 and sgRNA, especially in the case of hard-to-reach organs or when crossing the blood–brain barrier is necessary [44,45] (Table 1).

### 5.5. MCO-010

#### 5.5.1. What Exactly Is MCO-010?

MCO-010 is an optogenetic therapy based on an AAV2 viral vector that delivers the gene encoding multi-characteristic opsin—a protein capable of activating retinal bipolar cells in response to visible light. This therapy aims to restore light sensitivity in individuals with damaged photoreceptors, such as patients with retinitis pigmentosa. MCO-010 is administered as a single intravitreal injection and works independently of external devices to assist vision [64].

#### 5.5.2. Preclinical and Clinical Studies Phase 1 and 2

Rd10 mice carry a spontaneous missense mutation in the *Pde6b* gene, which encodes the cGMP phosphodiesterase 6B, causing a later onset and slower progression of retinal degeneration that resembles RP in humans. In rd1 and rd10 mouse models, characterized by genetically driven retinal degeneration, intravitreal injection of MCO-010 resulted in approximately 80% transduction of bipolar cells. The therapy was associated with slowed photoreceptor degeneration, preservation of normal retinal thickness, and improved neural connections to ganglion cells. Functional tests, including electroretinography, visual evoked potentials, optomotor response, and water maze tests, indicated restored visual responses in treated mice, lasting up to 26 weeks after therapy [64].

In an open-label Phase 1/2a study, four legally blind patients with RP, ranging from light perception to hand motion recognition, participated. Each patient received a single 100 µL intravitreal injection of MCO-010 following a short steroid regimen. All patients showed improvement in BCVA within 12 weeks, which remained stable through week 16 and then plateaued between weeks 31 and 52, likely due to patient dropouts and vitreous haze. Additionally, improvement in the visual field index was observed [65] (Table 1).

The Phase 2b RESTORE study, which was randomized and placebo-controlled, enrolled 27 patients with advanced RP. After 52 weeks of treatment, the primary endpoint was achieved—both the low-dose group showed an improvement in visual acuity of 0.382 LogMAR (*p* = 0.029), and the high-dose group showed an improvement of 0.337 LogMAR (*p* = 0.021) compared to the placebo. Moreover, patients in the high-dose group maintained and even increased their improvement at week 76, reaching 0.539 LogMAR (*p* = 0.001).

Functionally, about 89% of patients treated with MCO-010 showed an improvement of at least two levels in shape recognition tests under various light intensities or in mobility tests at week 52 of the study [64].

#### 5.5.3. Future Development and Direction of MCO-010

Based on the results of the RESTORE study, Nanoscope Therapeutics, which is a company focused on gene and optogenetic therapies for inherited retinal diseases, including RP, has indicated plans to submit a Biologics License Application (BLA) to the U.S. FDA in the second half of 2025. If approved, the MCO-010 therapy could become the first optogenetic treatment available to a wide range of patients with RP, regardless of the type of genetic mutation. For RP patients in Poland and Europe, FDA approval may facilitate additional clinical trials and potentially support regulatory pathways in the European market [66] (Figure 1) (Table 1).

#### 5.5.4. Side Effects of MCO-010

MCO-010 was generally tolerated, with no serious or severe adverse events related to the therapy observed in the studies, consistent with previous research. The most commonly reported adverse events included mild or moderate occurrence of cells in the anterior chamber of the eye and elevated intraocular pressure. No significant adverse events were noted in the treated groups, such as endophthalmitis, retinitis, retinal vasculitis, retinal vascular occlusion, or hypotony [66] (Table 1).

### 5.6. QR-1123

QR-1123 is currently under investigation as a potential therapy for autosomal dominant retinitis pigmentosa (adRP) linked to the *RHO* c.68 C > A mutation, which leads to a proline-to-histidine substitution at position 23. Approximately 15–25% of RP cases are autosomal dominant [2]. While over 20 genes have been associated with adRP, the *RHO* gene is the most frequently involved, accounting for 20–30% of cases [61]. This specific mutation is found primarily in the United States, where it affects an estimated 2500 to 3000 individuals. It causes a P23H substitution (proline-to-histidine at position 23) in the rhodopsin protein, leading to a misfolded protein and gradual photoreceptor degeneration [65] (Table 1).

QR-1123 is a mutation-specific antisense oligonucleotide (AON) designed to activate RNase H1 and selectively degrade mutant *RHO* mRNA containing the *P23H* variant, thereby reducing production of the toxic protein. Preclinical studies have shown that QR-1123 effectively lowers P23H rhodopsin levels and helps prevent retinal degeneration in both animal models and human cell systems of adRP [67] (Table 1).

The Aurora study (NCT04123626), started in 2019, called “A Study to Evaluate the Safety and Tolerability of QR-1123 in Subjects With Autosomal Dominant Retinitis Pigmentosa Due to the P23H Mutation in the *RHO* Gene”, sponsored by ProQR Therapeutics, is a first-in-human clinical trial designed to assess the safety, tolerability, and preliminary efficacy of QR-1123 in patients with adRP caused by the P23H mutation in the *RHO* gene. This active, not recruiting study includes up to eight cohorts receiving either a single intravitreal (IVT) injection in an open-label setting or repeated IVT injections every three months, evaluated in a randomized, double-masked, sham-controlled manner. All participants are monitored over a 12-month period, and dose escalation proceeds only after a safety review by an independent Data Monitoring Committee. The aim is to evaluate whether suppressing expression of the mutant P23H protein can preserve retinal structure and function in this genetically defined patient population [68] (Figure 1) (Table 1).

This study is a promising step toward personalized treatment in adRP, with the potential to address the underlying genetic cause in patients carrying the P23H mutation in the *RHO* gene.

### 5.7. SPVN06

SPVN06 is a mutation-agnostic investigational gene therapy developed by SparingVision for the treatment of rod–cone dystrophies, including RP. The therapy uses an AAV vector to deliver, via subretinal injection, two therapeutic components encoded by the *NXNL1* gene: the Rod-derived Cone Viability Factor (RdCVF), a neurotrophic factor that promotes cone metabolism, and the Rod-derived Cone Viability Factor Long form (RdCVFL), an antioxidant enzyme that protects cones from oxidative stress. Acting synergistically, these proteins aim to slow or halt cone photoreceptor degeneration, helping preserve central vision. SPVN06 does not target any specific mutation, making it potentially suitable for a broader range of RP patients. It is an important advancement given the current lack of approved therapies for genetically diverse RP populations. Preclinical studies in animal models, including *P23H* rhodopsin transgenic pigs, have shown that SPVN06 can help maintain cone structure and function. The therapy may also be applicable to other retinal diseases characterized by early rod loss, such as geographic atrophy in dry age-related macular degeneration [69,70] (Table 1).

PRODYGY (“Promising ROd–cone DYstrophy Gene TherapY”), a clinical trial sponsored by SparingVision (NCT05748873), is a multicentric Phase I/II trial. Its aim is to assess the safety, tolerability, efficacy, and life quality after one dose of subretinal injection of SPVN06 in the patients with rod–cone dystrophies (including RP) caused by the mutation in the *RHO*, *PDE6A*, or *PDE6B* gene [70] (Figure 1) (Table 1).

According to a recent press release from SparingVision (21 January 2025), the Phase I/II evaluating SPVN06 has progressed to its extension phase (Step 2), following favorable safety outcomes in the dose-escalation phase (Step 1). In total, nine patients received escalating doses of SPVN06 with good tolerability.

Phase II (step 2) of this controlled, randomized study, conducted across six clinical centers in France and the USA, will involve 24 patients with advanced intermediate-stage rod–cone dystrophy, randomly assigned to high-dose (*n* = 9), medium-dose (*n* = 9), or control, uninjected groups (*n* = 6). These results are based on publicly available company communications and have not yet undergone peer reviews [70,71] (Table 1).

**Table 1:** (a); (b) Summary of novel retinitis pigmentosa therapies.

(a)			
**Feature**	**Luxturna**	**OCU400**	**MCO-010**
Therapy Type	Gene replacement (*RPE65*-specific) [53]	Gene-agnostic (*NR2E3* network modulator) [55,56]	Optogenetic (mutation-independent) [64]
Target Gene/Mechanism	*RPE65* (biallelic mutations) [46]	*NR2E3* (network reprogramming) [55]	*MCO* gene (multi-characteristic opsin) [64]
Delivery Method	Subretinal injection [46]	Subretinal injection [55]	Intravitreal injection [65]
Eligibility	Only for patients with confirmed *RPE65* biallelic mutations [46]	Broad RP patients’ population [56]	All RP genotypes [64]
Clinical Trial Phase	Completed Phase 3 + approved [50]	Phase 1/2 completed, Phase 3 ongoing [57,58]	Phase 2b completed (RESTORE study) [64]
Efficacy Highlights	Improvement in functional vision (MLMT); enhanced light sensitivity (FST); better real-life mobility; durable efficacy; greater gains in younger patients [50,51,52]	Visual function preserved/improved in 100% of treated eyes; enhanced low-light visual acuity; stabilization/improvement in mobility performance (MLMT) [58]	Significant visual improvement in mobility tests in varying light conditions [64].
Adverse Effects	Retinal detachment; cataract; inflammation; ↑ intraocular pressure [48]	Mild events; surgery-related SAEs resolved without lasting effects [57]	Mild intraocular pressure ↑, anterior chamber cells [66]
Long-term Data	Available (up to 2.3 years, PERCEIVE) [49]	Available (2 years) [58]	Limited (short-term follow-up)
Regulatory Status	FDA + EMA approved [46]	EMA: ATMP status, FDA: EAP granted [59,60]	BLA planned H2 2025 [66]
Limitations	High cost; limited to *RPE65*; subretinal injection risk [48]	Still under investigation (not yet approved); limited long-time data; uncertain response across all genotypes; subretinal injection risk [58,59,60]	Awaiting approval; limited long-term data
(b)			
**Feature**	**CRISPR (general)**	**QR-1123**	**SPVN06**
Therapy Type	Gene editing (precise mutation correction) [44]	AON; allele-specific; RNase H1-activating [67]	Mutation-agnostic AAV-based gene therapy delivering two functional proteins encoded by the *NXNL1* gene [69,71]
Target Gene/ Mechanism	*RHO*, *CEP290*, etc. (mutation-specific) [63]	*RHO* gene with *P23H* mutation (c.68 C > A) [66]	Targets cone survival through delivery of RdCVF and RdCVFL [69,70]
Delivery Method	Viral/non-viral [44]	IVT injection (unilateral); single or repeated every 3 months [68]	Subretinal injection of AAV vector carrying RdCVF and RdCVFL genes [69]
Eligibility	Mutation-specific; under development [63]	Patients with adRP caused by the *P23H* mutation in the *RHO* gene [66]	Patients with rod–cone dystrophies, especially RP in intermediate stage [70]
Clinical Trial Phase	Preclinical and early clinical trials [63]	Phase I/II (Aurora Study, initiated in 2019); currently active but not recruiting [68]	Phase I/II clinical trial (PRODYGY) [70]
Efficacy Highlights	Some restored visual function in trials [45] Depends on gene; early results promising	Preclinical studies in adRP animal and human models demonstrated selective reduction of *P23H* rhodopsin and prevention of photoreceptor degeneration [67]	Preclinical studies in *P23H* rhodopsin transgenic pigs showed preservation of cone structure and function [69]
Adverse Effects	Off-target effects; immune response; DSB risks [44,45,63]	Not fully disclosed; ocular and non-ocular adverse events monitored over 12 months as primary outcome measures [68]	Phase I (PRODYGY) demonstrated good tolerability [71]
Long-term Data	Not available yet	Long-term human data not yet available; trial includes a 12-month follow-up period [68]	Not yet available; trial ongoing
Regulatory Status	Experimental	Investigational drug; not approved for clinical use yet [68]	Investigational drug; not approved for clinical use yet
Limitations	Precision/safety concerns, regulatory delay [44,45]	Mutation-specific therapy (effective only for *P23H* variant); mutation is rare and primarily found in the U.S. population (~2500–3000 individuals) [67]	Still in early clinical development; long-term efficacy and safety data pending; requires subretinal surgery

Signs: ↑—elevation.

## 6. Discussion

RP is an inherited, progressive retinal disease leading to gradual vision loss, narrowing of the visual field, and often blindness. The currently available treatments do not provide a complete cure; their main goal is to slow disease progression and improve patients’ quality of life. Supplementation with vitamin A palmitate may have neuroprotective effects but requires caution due to toxicity risks, and lack of standardization limits its widespread use. Additionally, components such as omega-3 fatty acids, lutein, and zeaxanthin exhibit anti-inflammatory and antioxidant properties that support retinal function [27,28].

Pharmacological therapies used to treat RP complications, such as CME, rely primarily on carbonic anhydrase inhibitors as a first-line treatment, with corticosteroids, VEGF inhibitors, and anti-inflammatory drugs serving as second-line therapies. Retinal prostheses, like ARGUS II, provide support for patients in advanced disease stages, but their use is limited by strict criteria and the risk of complications [32,34].

Modern methods such as hyperbaric oxygen therapy have shown potential in protecting photoreceptors but carry risks of complications and require further research. Stem cell therapies offer prospects for retinal regeneration, although clinical outcomes are currently inconclusive and associated with potential complications, such as retinal detachment [37,38,42,43].

A breakthrough in treating inherited retinal dystrophies, including some forms of RP, is gene therapy with Luxturna (voretigene neparvovec-rzyl), targeted at mutations in the *RPE65* gene. This FDA-approved therapy delivers a functional copy of the gene to retinal cells via an AAV vector, improving visual functions, especially dark adaptation and mobility in low light. Despite limitations such as invasive delivery, high cost, and limited scope, Luxturna represents an important advancement in gene therapy.

OCU400 therapy, independent of specific mutations, uses the *NR2E3* gene to restore retinal homeostasis and inhibit degeneration. Clinical studies have shown good tolerance and sustained vision improvement and mobility function over two years in patients with various mutations. Its ATMP status and FDA early access program may allow broad implementation in the US and Europe by 2027 [47].

CRISPR–Cas technology offers an innovative and precise approach to correcting genetic mutations underlying RP. Methods such as base editing and prime editing enable genome modification without introducing double-strand DNA breaks, increasing therapy safety. However, challenges related to off-target effects, mutagenesis, and efficient delivery of gene editors require further research and development.

MCO-010 is a novel optogenetic therapy that, through a fast, broadband, and highly sensitive opsin activated by ambient light, enables vision restoration by activating inner retinal layers, regardless of mutation type or disease stage. The therapy uses an AAV2 vector and mGluR6 promoter, ensuring effective expression in bipolar cells after intravitreal injection. Phase 1/2a clinical trials confirmed the therapy’s safety and dose-dependent improvement in visual acuity, with a good tolerance profile and minimal adverse effects [44,62]. The planned FDA registration submission for MCO-010 in the second half of 2025 opens the prospect of introducing the first optogenetic therapy to the broad market, potentially significantly impacting RP treatment worldwide, including in Poland and Europe [63,65].

Additionally, QR-1123 therapy is being investigated as a treatment for adRP associated with the *RHO* P23H mutation, the most common in the USA, accounting for 20–30% of adRP cases [2,11]. QR-1123 is an antisense oligonucleotide that selectively degrades mutant mRNA, reducing the production of the toxic protein. Preclinical studies have confirmed its efficacy, and the ongoing Aurora clinical trial is evaluating the safety and effects of the therapy [68]. This represents an important step toward personalized treatments for patients with adRP [11,17].

Another significant advancement is the gene therapy SPVN06, which is mutation-independent and uses an AAV vector for the subretinal delivery of two proteins encoded by the *NXNL1* gene (RdCVF and RdCVFL), which protect cone cells and support their metabolism, slowing degeneration and preserving central vision [69,70]. Preliminary PRODYGY studies (Phase I/II) demonstrated good tolerability in patients with various RP mutations, and Phase II is currently underway, offering hope for broad application of the therapy [69,70].

It is important to highlight that the reviewed paper demonstrates both the strengths and limitations of the present work. One of the main advantages of this review is its comprehensiveness—the authors present both traditional supportive therapies and the latest advancements in gene therapy, optogenetics, and CRISPR technology. The inclusion of a wide range of therapeutic strategies—from pharmacological treatments and supplementation to cell therapies and technological innovations—provides a complete picture of the available options. The review also incorporates up-to-date clinical trial data, which enhances its credibility and relevance. Furthermore, the paper emphasizes the importance of personalized and mutation-independent therapies, addressing the real clinical needs of RP patients. An additional strength is the discussion of the potential applicability of these emerging therapies in Europe, including Poland, which increases the practical value of the work.

However, the review is not without its limitations. As a narrative review, it does not include detailed statistical analyses of treatment efficacy or direct comparisons between different therapies. It also lacks a critical assessment of the costs and accessibility of the treatments across various countries, which limits its usefulness in health system analyses. Some of the therapies described are based on data from early-phase clinical trials, which means that certain conclusions remain preliminary and require further validation in broader clinical practice. Moreover, ethical and social considerations related to the implementation of new gene and optogenetic therapies are only marginally addressed. This review primarily focuses on biological and technological approaches, with less attention given to rehabilitation and psychological support—important components of comprehensive care for patients with RP.

## 7. Conclusions

Retinitis pigmentosa remains an incurable but increasingly manageable disease thanks to recent therapeutic advances. Traditional approaches such as vitamin supplementation and pharmacological treatment help alleviate symptoms but offer limited long-term benefits. Novel gene therapies, including Luxturna and OCU400, show potential in restoring retinal function and slowing down the degeneration process, although their application is limited by factors such as high cost, invasive delivery, and genetic specificity. Optogenetic treatments like MCO-010 present a mutation-independent solution, showing promising safety and efficacy results. Meanwhile, CRISPR-based genome editing offers a precise and innovative direction for future therapies, though it still faces challenges related to delivery efficiency and safety concerns.

In summary, the development of gene therapies, optogenetic approaches, and innovative treatment methods for RP offers real hope for improving visual function and patients’ quality of life. However, each of these approaches requires further clinical trials, long-term safety monitoring, and the development of treatment standards to enable their safe and effective use in clinical practice.

## Data Availability

No new data were created or analyzed in this study. Data sharing is not applicable to this article.

## Abbreviations

AAV	Adeno-Associated Virus
adRP	Autosomal dominant retinitis pigmentosa
AMD	Age-related macular degeneration
Anti-VEGF	Anti-Vascular Endothelial Growth Factor
AON	Antisense oligonucleotide
ATMP	Advanced Therapy Medicinal Product
BCVA	Best-corrected visual acuity
BLA	Biologics License Application
CME	Cystoid Macular Edema
CPC	Chromatic pupillometry
CRISPR	Clustered Regularly Interspaced Short Palindromic Repeats
CRISPR–Cas9	CRISPR-associated protein 9
crRNA	CRISPR RNA
DSBs	Double-strand breaks
EAP	Expanded Access Program
EMA	European Medicines Agency
ERG	Electroretinogram
FAF	Fundus autofluorescence
FDA	Food and Drug Administration
FST	Full-field light sensitivity threshold testing
gRNA	Guide RNA
IOL	Intraocular lens
IRD	Inherited retinal disease
IVT	Intravitreal
LCA	Leber congenital amaurosis
MLMT	Multi-luminance mobility test
NR2E3	Nuclear Receptor Subfamily 2 Group E Member 3
pre-mRNA	Precursor mRNA
PRPF31	Pre-mRNA processing factor 31
RdCVF	Rod-derived Cone Viability Factor
RdCVFL	Rod-derived Cone Viability Factor Long Form
RHO	Rhodopsin Gene
ROS	Rod outer segment
RP	Retinitis pigmentosa
RPE	Retinal pigment epithelium
RPE65	Retinal pigment epithelium-specific 65 kDa
SAEs	Serious adverse events
sgRNA	Single-guide RNA
TEAEs	Treatment-emergent adverse events
tri-snRNP	Tri-small nuclear ribonucleoprotein
TWIN-PE	Twin prime editing
USH1	Usher syndrome type I
USH2A	Usher syndrome type IIA
USH3	Usher syndrome type III
